# Disengaged response behavior when the response button is blocked: Evaluation of a micro-intervention

**DOI:** 10.3389/fpsyg.2022.954532

**Published:** 2022-11-03

**Authors:** Lothar Persic-Beck, Frank Goldhammer, Ulf Kroehne

**Affiliations:** ^1^DIPF | Leibniz Institute for Research and Information in Education, Frankfurt, Germany; ^2^Centre for International Student Assessment (ZIB), Frankfurt, Germany

**Keywords:** log data analysis, rapid responding, rapid guessing behavior, rapid omission, test taking behavior, process data analysis, finite state machine

## Abstract

In large-scale assessments, disengaged participants might rapidly guess on items or skip items, which can affect the score interpretation’s validity. This study analyzes data from a linear computer-based assessment to evaluate a micro-intervention that blocked the possibility to respond for 2 s. The blocked response was implemented to prevent participants from accidental navigation and as a naive attempt to prevent rapid guesses and rapid omissions. The response process was analyzed by interpreting log event sequences within a finite-state machine approach. Responses were assigned to different response classes based on the event sequence. Additionally, *post hoc* methods for detecting rapid responses based on response time thresholds were applied to validate the classification. Rapid guesses and rapid omissions could be distinguished from accidental clicks by the log events following the micro-intervention. Results showed that the blocked response interfered with rapid responses but hardly led to behavioral changes. However, the blocked response could improve the *post hoc* detection of rapid responding by identifying responses that narrowly exceed time-bound thresholds. In an assessment context, it is desirable to prevent participants from accidentally skipping items, which in itself may lead to an increasing popularity of initially blocking responses. If, however, data from those assessments is analyzed for rapid responses, additional log data information should be considered.

## Disengaged response behavior in blocked item response: Evaluation of a micro-intervention

Disengaged response behavior in the assessment context means that participants are not motivated to respond to questions with maximum performance according to their ability. Thus, disengagement is a threat to the validity of test score interpretation (Wise, 2015, 2017). This study analyzes data from a computer-based assessment, containing a micro-intervention that blocked the possibility to navigate to the next item for 2 s after items got loaded (2-s-blocking). The developer’s rationale for the intervention was to prevent participants from accidentally responding by clicking on the next button immediately after an item is loaded. It was also expected that the 2-s-blocking would interfere with rapidly given responses by disengaged participants and that it might even prevent such response behavior. However, there is no empirical data about the effects of such a feature.

Disengaged test takers may rapidly guess an answer or rapidly omit an item (Wise and Gao, 2017; Wise, 2019). Rapid guessing will be used as a term for response behavior of participants who do not take sufficient time to solve an item and instead quickly select a response option at random or apply a fixed response pattern. Rapid omission will be used as a term for response behavior of participants who quickly skip an item without giving a response at all. The term rapid responses is used below to refer to both behaviors, rapid guesses, and rapid omissions. Solution behavior, in turn, is used as a term for behavior directed toward solving tasks according to the ability in question (Schnipke and Scrams, 1997).

Response time data with a certain amount of rapid responses will typically show a bimodal response time distribution (Schnipke, 1995; Wise, 2017). In *post hoc* analyzes, responses can be classified as solution behavior or rapid responses according to their response time, by either setting response time thresholds(see Wise (2019) for an overview) or by mixture modeling (see Ulitzsch et al. (2020) for an overview).

In computer-based testing, interactions with the user interface can be captured by log events, for example, if a participant clicks on a response option of a multiple-choice item. Log events usually come with timestamps, from which response times can be derived. Besides item responses, response times became an additional source for interpreting test results (e.g., van der Linden, 2007). But even more than using log events for extracting responses and response times, log data and thus the response process itself gets more and more into the focus of scientific interest (Goldhammer and Zehner, 2017). A comprehensive approach to log data analyses is offered by Kroehne and Goldhammer (2018), who used the concept of finite-state machines (FSM) from computer science to conceptualize the algorithmic extraction of features from log data. The response process can be in a finite set of states within this concept, like reading or working. The human-computer interaction can be captured by log events marking transitions between these states. For example, selecting a response option on a multiple-choice item can be seen as a transition from the reading state to the answering state. Different response behaviors cause different sequences of states of the response process, which can be described using n-grams. With this approach, n-grams describing state-sequences can be defined and interpreted as behavior in reaction to the micro-intervention.

This study uses the finite-state approach to analyze behavior in the context of the 2-s-blocking. Solution behavior, rapid guessing, rapid omission, and accidental clicks will be distinguished by sequences of states. Additionally, response time thresholds are used to cross-check this approach.

### Response process model

For items with closed response formats in computer-based tests, a minimal set of log events resulting from user interactions would be the log events representing the selection and deselection of response options (answer-change events) and the events resulting from the navigation between items or, if required resulting from response confirmation. The events that correspond to a particular item typically start with an event that indicates that the item was loaded, followed by some idle time, followed by one or more interactions with the response options, and, if required, followed by some button click indicating response confirmation. On an interpretative level, the idle time before the first interaction potentially represents behaviors like reading or, more broadly, information processing if items provide image-or video material. Also, the solution process will start in that period and possibly result in a response selection. If a test requires an explicit response confirmation, the period between response selection and response confirmation could include metacognitive processes of reevaluating the given answer.

The actual response process might be much messier. Within the FSM approach (Kroehne and Goldhammer, 2018), the conceptualization of the response process can only be as detailed as response events are available. Additionally, states do not have to be ordered. They can overlap or alternate several times. Participants could select a response option before they processed all alternatives or go back to reading the instructions while trying to select the correct response. Some persons may review their response before confirming it, while confirmation equals response selection for others.

However, describing the response with a finite set of states and clearly defined transitions between them has certain advantages. It has no additional assessment effort, and log events are a direct product of behavior in contrast to self-reports on behavior. Deriving interpretable states from mere log events has the advantage that the sequence and duration of those states can be used to validate their interpretation. For example, a recent study by Sahin and Colvin (2020) used frequencies of log events to enhance response time threshold estimation for rapid guessing. The study showed that behavior such as rapid guessing could be inferred from log events. Concerning the 2-s-blocking, it is not only of interest which and how many log events are in the data, but also in which sequence they occurred, how much time has passed between them, and how they can be interpreted in a meaningful way. For example, what happened before and after the micro-intervention and how much time participants spent in certain sections of the response process would be interesting.

### Response time-based detection of rapid responding

Under the assumption that response times in a data set with rapid responses originate from two distinct response time distributions, a time threshold can be defined between the two distributions. Responses below the threshold are classified as rapid responses, and responses above the threshold as solution behavior. Besides the shape of the distribution, response accuracy in the form of average scores per time segment (Lee and Jia, 2014) or cumulative score per time segment (Guo et al., 2016), or response information in the form of item score with person total score correlation (Wise, 2019) can be used for setting response time thresholds.

For a manageable number of items, response time thresholds can be set by a visual inspection (Wise, 2006, 2019) of the response time distribution. Raters determine the intersection of the response time distributions. Wise (2019) introduces the Change in Information and Accuracy method (ChIA), which adds accuracy and item information to the visual inspection of response times. It can be seen as a combination of different methods for response time threshold estimation.

For larger item pools, visual inspection becomes unhandy. Another common approach is to set a fixed response time threshold, whereby 3-s is a rule of thumb (Kong et al., 2007; Lee and Jia, 2014), depending on the reading time that items require. Since reading times may differ between items or item formats, thresholds can be adapted per item. They can also be set relative to the average response time (Normative Threshold method, Wise and Ma, 2012, April), or other measures like response accuracy above guessing chance can be used for automated threshold estimation (Goldhammer et al., 2016). However, there are no mandatory criteria for the existence of a bimodal distribution, and that an automatically set threshold value is placed at the intersection between the two assumed distributions.

Model-based approaches are another possibility for response classification (Schnipke and Scrams, 1997; Ulitzsch et al., 2020; Deribo et al., 2021). Alternative to dichotomous classification, a probability of a response being a rapid response can be estimated. However, the model fit might not always be satisfying, and mixture modeling can have practical issues. The data may not fit the prerequisite, or convergence may fail, especially for smaller samples.

### Constructs related To test engagement

The relevance of test results to participants is often assumed to impact test-taking engagement (Wise and Kong, 2005; Wise, 2017). If test results have little consequence to participants, the conditions are considered low-stakes, and participants are expected to be more prone to disengagement. If test results have more severe consequences for participants, the conditions are considered high-stakes, and disengagement is expected to occur less frequently.

Besides high-and low-stakes conditions, studies have found other factors related to rapid responding. Cognitive skills, gender, primary language, and age are investigated as covariates of test-taking engagement by Goldhammer et al. (2016) and Goldhammer et al. (2017). A study by Lindner et al. (2019) showed a relation between the onset of rapid guessing and general cognitive ability and working memory capacity. Goldhammer et al. (2017) found a negative relation between effort and item difficulty and therefore deduced a positive relation between ability and effort. A study by Deribo et al. (2021) found a negative relation between ability and the frequency of rapid guesses. In a study by DeMars et al. (2013) and in a study by Setzer et al. (2013), female participants showed more effort. However, Goldhammer et al. (2017) and Lindner et al. (2019) could not find a relation between gender and test-taking effort. If the language of the test is not the participant’s native language, the results of Goldhammer et al. (2017) suggest that the effort is negatively affected. A negative relation between effort and age was found in one out of two domains investigated by Goldhammer et al. (2017).

### The present study

#### MYSKILLS

The 2-s-blocking was implemented in the MYSKILLS assessment program, a standardized and proctored computer-based assessment that captures practical occupational knowledge. Items are presented one at a time, without the possibility to navigate backward. The computer-based assessment form allows for a large-scale application but may also bring pitfalls for the target population. Participants who are not particularly adept at using computers may commit errors during operation. Also, a delay in loading a new page may cause participants to click twice on the next button and thus actually skip an item. Therefore, the assessment’s developers wanted to prevent accidental answer giving. Additionally, the assessment’s developers assumed that the 2-s-blocking could hinder rapid response behavior resulting from disengagement. Given the text length of the shortest items, 2-s were chosen as a global value. Although theoretical considerations about the 2-s-blocking were made, its implementation was not meant as a theoretically well-founded intervention. This study analyzes data from the assessment program to evaluate the assumptions about the intervention.

MYSKILLS was standardized on a sample of German vocational students, to whom test results had no practical implications. Consequently, the test conditions can be described as low-stakes. A sample from the instrument’s standardization will be analyzed, as disengagement is thought to be more prominent under low-stakes conditions (Wise and Kong, 2005; Wise, 2017). Beyond that, data from the regular operations of MYSKILLS is used in this study. In regular operations, the test results are used for the labor market placement process and applications to employers. Consequently, the test conditions can be described as high-stakes. The two conditions allow to investigate the expected differences in rapid responses for high-and low-stakes and to evaluate if the behavior in reaction to the 2-s-blocking differs in a real live assessment context.

#### FSM model of the blocked response

The finite-state machine approach was chosen to conceptualize the response process. States can be used to form an interpretable layer from log events resulting from human-computer interaction. Their sequence and their temporal dimension could be used to distinguish and interpret different response behaviors.

**Figure 1:**
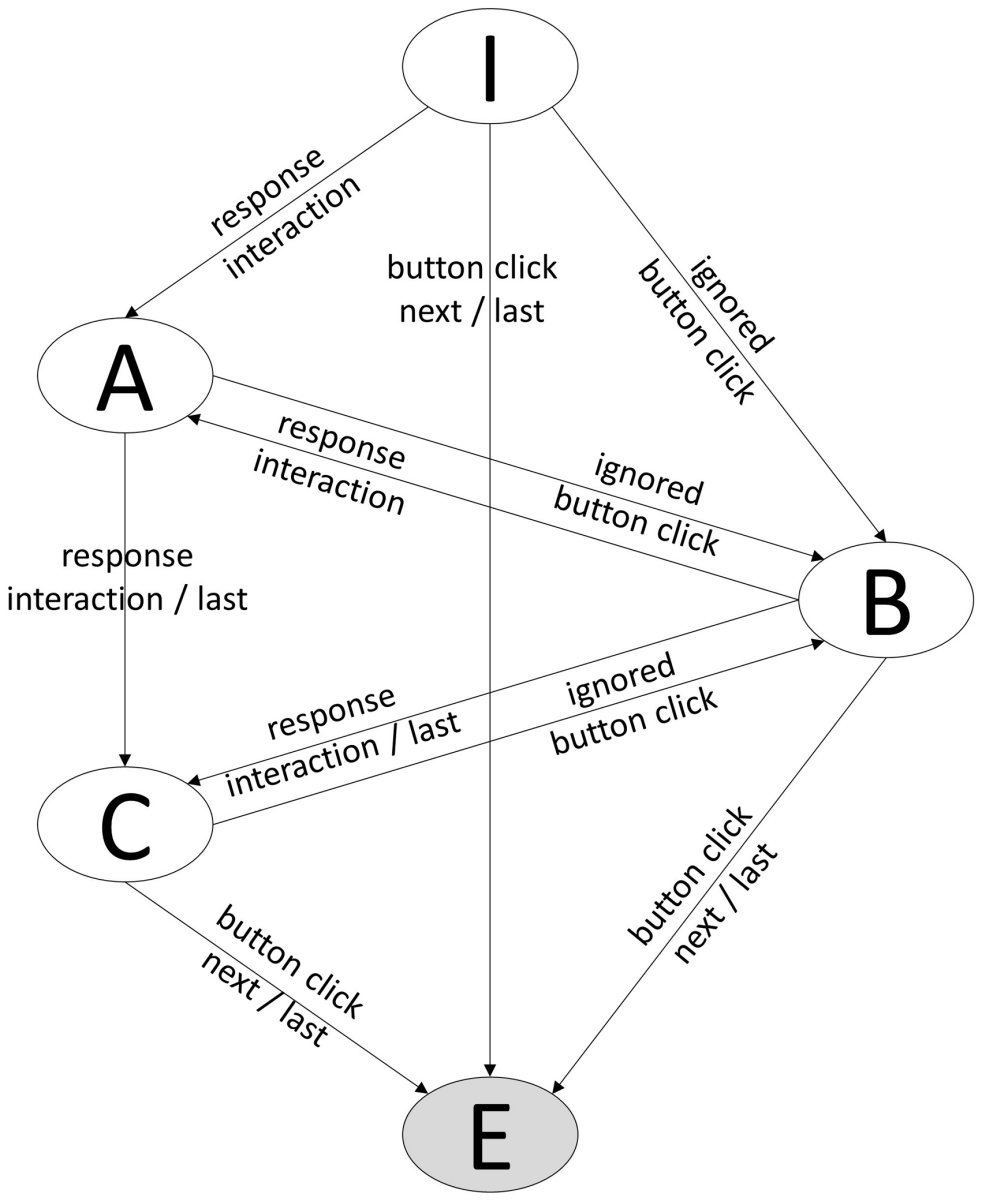
Finite state model of the response process. I, Information processing state; A, Answering state; C, Confirmation state, B, Blocked state; E, End-state.

For the present study, the following states of the response process are distinguished: information processing (I), answering (A), response confirmation (C), blocked state (B), and end-state (E). For that purpose, the transitions between states are identified by observed log events. Information processing (I) would be the starting point when items are loaded. Selecting or moving response options can be used to identify the transition to the state answering (A). The last interaction with any response element of a particular item marks the transition to the response confirmation state (C). It should be noted that in the case of single-choice items, the first interaction with response elements can equal the last interactions. In this case, the information processing state will directly be followed by the response confirmation state. Finally, the click to confirm the response will lead to the end-state, and the item gets unloaded. Within the MYSKILLS software, a special event was thrown when a click on the next button was not accepted due to the time limit, called ignored button click. The event is used as the trigger for a transition from the information processing (I) or answering (A) state to the blocked state (B). The complete state model for the response process for the MYSKILLS-items is shown in Figure 1.

#### Response classes described by N-grams

Figure 1 includes all possible states and transitions. The response process for a particular item will show a distinct sequence of those states that can be described using n-grams. Different response behavior will result in different n-grams. For example, if a participant clicks on the next button within the first 2-s, the n-gram will contain a blocked state. An ignored button click indicates rapid responding under the assumption of a fixed time threshold of 2-s. However, the response process is not finished at this point, and it is crucial what happens afterward. A participant could simply click again on the next button until the item finally gets unloaded. This could increase the total time of the response process by a few seconds but still would strongly indicate disengaged response behavior. In contrast, a participant might change or provide a response and spend a considerable amount of time on the item. In that case, the response is presumable a result of solution behavior. The ignored button click might result from an accidental click, or the micro-intervention led to a behavioral change. Consequently, under the *a-priori* assumption that 2-s-blocking captures rapid responding, state-sequences can be used to distinguish between solution behavior, accidental clicks, prevented rapid responses, and rapid responses. Table 1 shows the responses classes and the associated state-sequences defined for this study. The n-grams are theory-defined and expected in the data. Their prevalence must be determined empirically.

**Table 1:** Response classes.

	Group	Class	State-sequence	N-gram of states
1	No ignored button click	Regular response	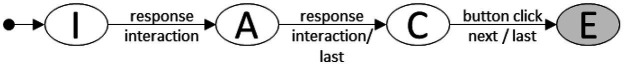	IACE
2		Omission	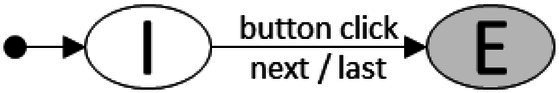	IE
3	Returner	Accidental click/prevented omission	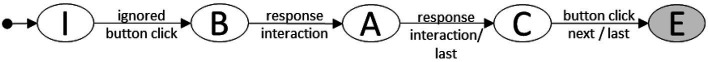	IBACE
4		Prevented guess	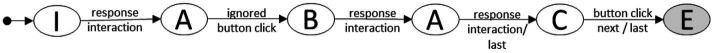	IABACE
5	Non-returner	Rapid guess	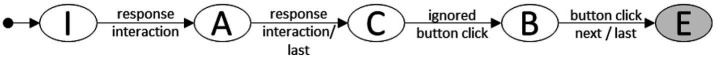	IACBE
6		Rapid omission	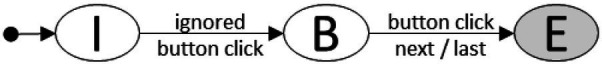	IBE

I, Information processing state; A, Answering state; C, Confirmation state; B, Blocked state; E, End-state.

#### Response times

A straightforward approach to derive response times is to take the time on task. In terms of log data, the time difference between the loading event of an item and an event indicating the end of the response process would give this information. But with the 2-s-blocking, participants might be prevented from completing the response process and respond multiple times. Moreover, the first and the last attempt to respond could stem from different motivations if a participant’s engagement changes through the micro-intervention. Following the described response classes, the behavior after the micro-intervention is decisive for the classification as solution behavior because only the final response is taken for ability estimation. Participants who clicked on the next button by accident or participants who got re-engaged after the 2-s-blocking should spend sufficient time after the 2-s-blocking for the response to be classified as solution behavior. In contrast, disengaged participants showing rapid response behavior who did not reengage should spend only a short amount of time on task after the 2-s-blocking.

### Research goal: Evaluation of the 2-s-blocking

The 2-s-blocking was implemented to prevent accidental skipping items, and it is expected to interfere with rapid responding. The response process will be analyzed with the finite-state machine approach. Responses from the two data sets will be classified using six distinct n-grams of possible state-sequences (Table 1). Additionally, response time-based methods for the *post hoc* detection of rapid responding will be applied to the data. If the response was in the blocked state at some point, only the time period after the last blocked state is considered. Response time-threshold-based classification of solution behavior and classification based on state-sequences will be compared across conditions and regarding the covariates ability, gender, primary language, and age. Covariates of test-taking engagement should have the same relation with rapid responses classified through state-sequences and rapid responses classified through response time-based methods.

The prevalence of state-sequences can be used to investigate how often the response classes occur in practice and how often participants return or not to interact with the item after the 2-s-blocking. This leads to the first research question:

1. What is the prevalence of disengagement-related response behavior operationalized as state-sequences for a task design using a micro-intervention of a 2 s blocked next button?

The response time-based classification can be considered a reference method to analyze the extent to which the 2-s-blocking interferes with rapid responding. Because the response time-based thresholds will likely not equal precisely 2 s, not all responses below the threshold will have a blocked state. Conversely, rapid responses classified by state-sequences may have response times above response time threshold values because there is no time limit for the response process after the 2-s-blocking. This leads to the second research question:

2. How effective is the 2-s rule implemented in the micro-intervention in identifying rapid responses compared to response time-based methods?

Finally, evidence for the validity of the interpretation of the response classes is examined. Rapid responses classified through state-sequences should have response times distinguishable from solution behavior (Schnipke, 1995; Wise, 2017) and scores around the guessing chance (Lee and Jia, 2014; Guo et al., 2016). Accidental clicks and prevented guesses should have response times and scores equal to solution behavior. The onset of solution behavior should not be before 2-s if the *a-priori* assumption is true that triggering the blocked state results from rapid responding or accidental clicks. Furthermore, the practical use of the state-sequence classification of rapid responses would be limited if participants learn to avoid clicking on the next button while it is blocked, and as a result, rapid responses can no longer be detected by the blocked state. Therefore, learning effects will be analyzed through the relation of response time-based classification and state-sequence-based classification in the course of the assessment. At last, evidence for the interpretation of both classification systems can come from the expectations about test-taking engagement for the context of high-and low-stakes and the person variables. This leads to the third research question:

3. Is there validity-evidence to interpret responses classified as rapid responding using the 2-s rule implemented in the micro-interventions as indicators of low test-taking effort?

## Materials and methods

### Sample

Two samples were used for this study. The first sample, representing low-stakes conditions, is a subsample from the standardization of the MYSKILLS assessment of vocational students in Germany for the test on the vendor profession and the test on the motor vehicle mechatronics technician. The data included *n* = 147 participants for the vendor, of which *n* = 73 were female, and *n* = 157 for the motor vehicle mechatronics technician, of which *n* = 2 were female. The second sample, representing high-stakes conditions, is a subsample from the regular operations of the MYSKILLS assessment for the test on the vendor profession and the test on the motor vehicle mechatronics technician from the years 2017 to 2019 in German. The data included *n* = 972 participants for the vendor, of which *n* = 711 were female, and *n* = 279 for the motor vehicle mechatronics technician, of which *n* = 13 were female.

### Instrument

MYSKILLS was developed for the German working agency with the help of the Bertelsmann Foundation. The target population is low-skilled workers without a formal qualification but working experience and persons with foreign qualifications that are not officially recognized in Germany. Participants can be tested in one of 30 professions. A battery of items subdivided into the main areas of expertise for each profession was developed. Participants received test results for each area. Items are illustrated with videos and images and presented in various closed response formats. Trained professionals proctor the test administration. The typical duration is about four hours with a fifteen minutes break.

Professions within the MYSKILLS assessment program can be summarized into the broad categories of craft professions and service professions. To obtain a representative extract from the MYSKILLS data, we choose one frequently used test from each category, the vendor and the motor vehicle mechatronics technician. The test for the vendor includes 141 scored items, and the test for the motor vehicle mechatronics technician includes 145 scored items.

Participants will see one item at a time and confirm their response by clicking on the next button. Backward navigation is not possible and thus items cannot be revisited. Participants are told that the item will be considered incorrect if they do not select an answer. An item can have more than one correct response, and the test allows for partially correct responses. The partial credit model [PCM; Masters, 1982] is used for ability estimation. According to the assessment’s developers, each area of expertise is estimated with an independent IRT-model.^1^ Item parameters for individual ability estimation are delivered with the assessment.

The MYSKILLS-tests use four different types of items: multiple-choice, image-maps, sort items, and associate items. In a part of the multiple-choice items, participants can select more than one response. For image-map items, participants have to select one or more areas on an image to answer an item. For example, the image could show a toolbox, and participants were asked to select the appropriate tools for a given task. A series of working steps must be put in the correct order for sort items. The participants are instructed that the initial order is not the correct answer. Participants are asked to match technical terms and images for associate items *via* drag and drop. The item types differ in average guessing chances, ranging from 0.28 to 50.00% (M = 15.62, SD = 10.43). Single Choice items have the highest guessing chances, while sort and associate items have very low guessing chances, making correct guesses rather unlikely.

The 2-s-blocking disabled the functionality of the next button. When participants hovered over the next button before two seconds elapsed, a prohibition sign indicated that the button was disabled.

### Data analysis

All computations and plots for the statistical analysis were done using R (R Core Team, 2021).

#### Response classes

For each response of a person to an item, the state-sequence was derived from the log data. Responses were then classified according to the resulting n-grams described in Table 1. Log event sequences 1 and 2 were grouped as responses with no ignored button click, sequences 3 and 4 were grouped as returners, and sequences 5 and 6 were grouped as non-returners. Additionally, the number of ignored button click events was calculated for each response. Six responses from the rapid guess class had no answer selected when the next button was clicked. Four of them were given on sort-items. The log files revealed that the distractor was not moved far enough and returned to the original position. For the other responses, a distractor of a choice item was selected and deselected within Milliseconds with three and eight ignored button clicks afterward. Those responses were counted as rapid guesses. 520 responses from the omission class and two from the accidental click class had deselected response options.

#### Response times

Response times were calculated as the difference between the timestamp of the click on the next button and the item-loaded event. If the log data of the response contained ignored button clicks, the time difference between the first click on the next button and the item-loaded event was subtracted from the time difference between the last click on the next button and the item loaded event. In terms of state-sequences, either the beginning of the information processing state or, if present, the beginning of the blocked state was taken as a starting point, and the beginning of the end-state was taken as an endpoint for the response time calculation.

#### Response time threshold estimation

**Figure 2:**
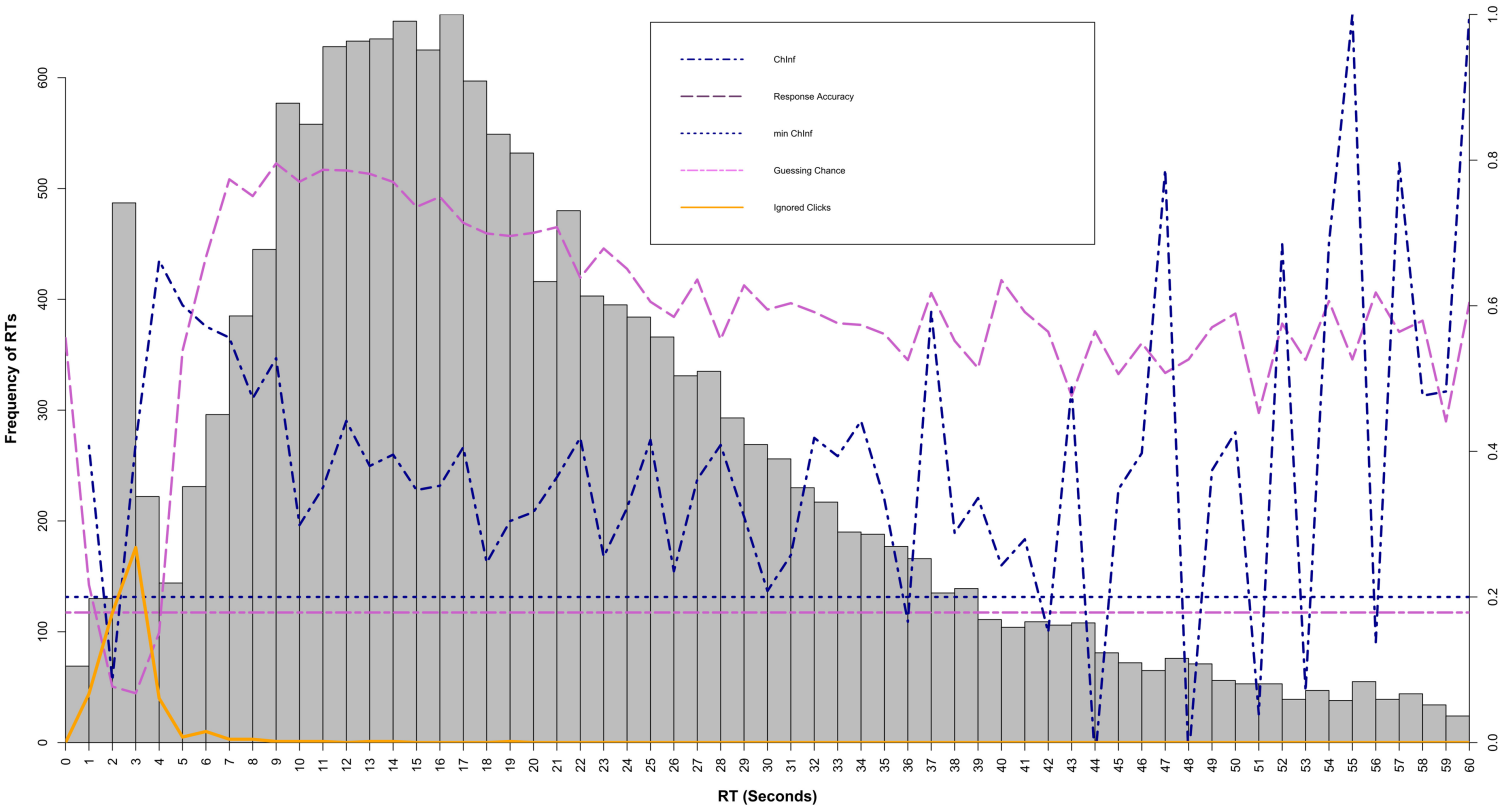
Visual inspection graph for the ChIA Method, Supplemented by the distribution of accepted responses with ignored button clicks classified as rapid responses. RT, response time; ChInf, change in information; min ChInf, the criterion for the ChInf method for the start of solution behavior. The histogram refers to the left *y*-axis. Ignored clicks corresponds to frequencies on the left *y*-axis. ChInf and min ChInf corresponds to correlation values on the right y-axis. Response accuracy corresponds to response accuracy values on the right *y*-axis. Guessing chance corresponds to the probability of guessing on the right *y*-axis.

The response time threshold estimation was oriented to the ChIA method (Wise, 2019). The ChIA method was chosen because it incorporates accuracy and information criteria and can be seen as a broad-spectrum approach. In addition, a visual inspection should ensure that bimodal distributions are indeed present and that the threshold is plausible. Due to the large item pool, item-level thresholds are not practical. A summary at the item type level was chosen as the coarsening level. Different item types can be assumed to have different numbers of rapid responses (Wise and Gao, 2017). There is a possibility that not all responses show a bimodal response time distribution at the item level, even if it is found at the item type level. This was found to be acceptable for comparison with state-sequence-based classification, as it leads to over-classification by the ChiA method in case of doubt. Therefore, thresholds are summarized by item type for each profession in each data set, resulting in 16 thresholds to be estimated. Response times for the four item types rounded to one second time segments were plotted in histograms and presented to two independent raters. According to the ChIA method (Wise, 2019), the plots included a line for the proportion of correct responses for each time segment, a dotted line for the guessing chance, a line for the correlation of scores from the time segment with the person level overall scores and a dotted line for the 0.2 correlation criterion (see Figure 2). Raters were advised to set the threshold at the average value between the first sustained increase of the proportion of correct responses above the guessing chance, the first sustained increase of the score correlation above 0.2, and the midpoint of the time segment with the lowest frequency between the two modes. After the rating, the average value between the two raters was used as the threshold value. Responses equal to or above the threshold value were classified as solution behavior, responses beneath as rapid responses.

To examine how item-type-level coarsening affects the classification of rapid responses, the NT10 method (Wise and Ma, 2012, April) was also applied, once on item-level and once on item-type-level. The NT10 method directly depends on mean response times and mean response times did not seem to be a sufficient measure, since the distribution of response times for rapid responses might be more homogeneous between items than the distribution of response times for solution behavior. However, if a bimodal response time distribution is given, responses below the threshold should be majority true fast responses. Therefore, sensitivity and specificity for item-type-level against item-level were used as indicators.

#### RTE and RTF

Once a threshold is set, and responses are classified accordingly, the amount of solution behavior per person and per item can be expressed by two indices: Response time effort (RTE) is an index developed by Wise and Kong (2005) that reflects the proportion of solution behavior for a person. In contrast, response time fidelity (RTF) (Wise, 2006) reflects the proportion of solution behavior for an item. RTE and RTF will be calculated for response time-based classified responses as well as for state-sequence-based classified responses. This will be done on test-level, but also for each area of expertise to calculate the correlation with the ability that is estimated for each area of expertise.

## Results

### Prevalence of the state-sequence classes

For 98.92% of all items, at least one response with ignored button clicks was present in the data. Two items from the vendor standardization data and four items from the motor vehicle mechatronic standardization data had no ignored button click at all. The median of ignored button clicks per scorable item was Md = 14.00. Values for items with ignored button clicks ranged from 1 to 98 regarding each item per data set. 18.55% of all persons under high-stakes conditions and 43.42% under low-stakes conditions had at least one ignored button click event throughout all responses. The median of ignored button clicks for persons with ignored button clicks was 2, ranging from 1 to 902. The 90% percentile was 38.40. The log data of the individuals with the five highest values (902, 605, 432, 388, 232, and 220) were reviewed and found to be plausible.

The percentage of responses assigned to each state-sequence class is shown in Table 2. N-grams 2–6 did occur relatively more often under low-stakes conditions, χ^2^ (5, *N* = 39,279) = 36328.16, *p* < 0.001. Rapid responses were more often assigned to the rapid omit class than to the rapid guess class. Rapid guesses occurred more frequently in choice items than in other item types. 10.09% of responses with a blocked state in the high-stakes condition and 7.84% of responses with a blocked state in the low-stakes condition with a blocked state were assigned to the returner group.

**Table 2:** Prevalence of state-event-sequences as absolute number and as percentage of total responses.

Stakes	Item type	Regular response	Omission	Accidental click	Prevented guess	Rapid guess	Rapid omission
High							
	Choice	136,812 (98.68%)	1,662 (1.20%)	12 (0.01%)	5 (0.00%)	12 (0.01%)	139 (0.10%)
	Sort	10,792 (93.33%)	745 (6.44%)	1 (0.01%)	0 (0.00%)	0 (0.00%)	25 (0.22%)
	Associate	7,841 (94.32%)	453 (5.45%)	3 (0.04%)	0 (0.00%)	0 (0.00%)	16 (0.19%)
	Imagemap	5,736 (95.46%)	268 (4.46%)	1 (0.02%)	0 (0.00%)	0 (0.00%)	4 (0.07%)
	Combined	161,181 (97.97%)	3,128 (1.90%)	17 (0.01%)	5 (0.00%)	12 (0.01%)	184 (0.11%)
Low							
	Choice	30,835 (95.89%)	654 (2.03%)	32 (0.10%)	13 (0.04%)	290 (0.90%)	331 (1.03%)
	Sort	3,153 (92.44%)	188 (5.51%)	5 (0.15%)	1 (0.03%)	10 (0.29%)	54 (1.58%)
	Associate	2,207 (94.76%)	86 (3.69%)	4 (0.17%)	0 (0.00%)	3 (0.13%)	29(1.25%)
	Imagemap	1,274 (92.05%)	66 (4.77%)	9 (0.65%)	0 (0.00%)	2 (0.14%)	33 (2.38%)
	Combined	37,469 (95.39%)	994 (2.53%)	50 (0.13%)	14 (0.04%)	305 (0.78%)	447 (1.14%)
Combined							
	Combined	198,650 (97.47%)	4,122 (2.02%)	67 (0.03%)	19 (0.01%)	317 (0.16%)	631 (0.31%)

### Response times

**Figure 3:**
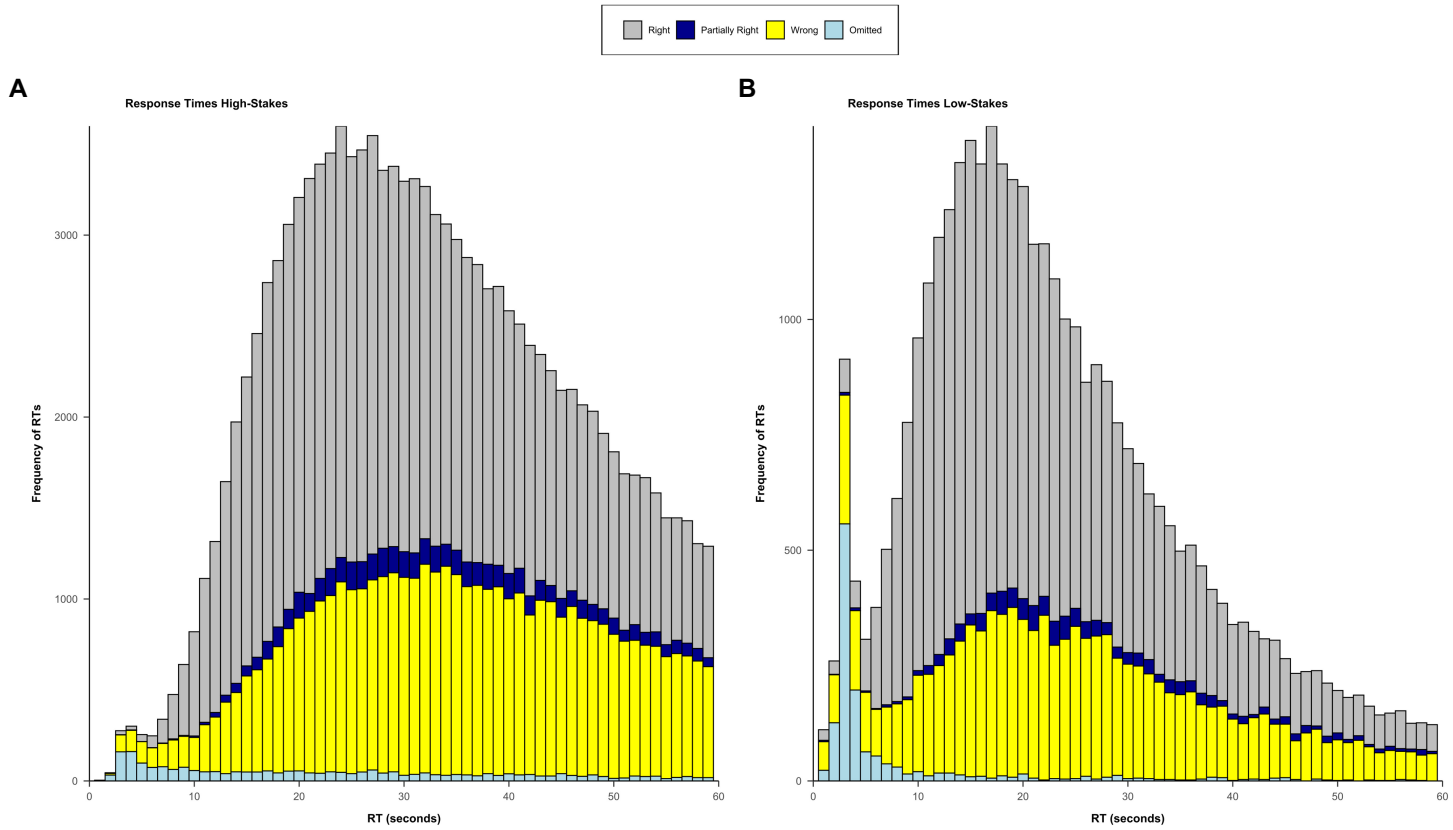
Response times per score (right, partially right, wrong, omitted) for the high- and the low-stakes data. RT, response time.

Figure 3 shows the response time distribution for each data set across all items per score. Both conditions, high-stakes, and low-stakes, show a bimodal response time distribution, whereby the peak is more pronounced for low-stakes. Additionally, the average response time is higher for the high-stakes group. Omitted responses are positively skewed but can continuously be found for higher response time values. The mean time difference between the first blocked response and the final, accepted response was 20.14 s for the returner group and 3.09 s for the non-returner group.

**Figure 4:**
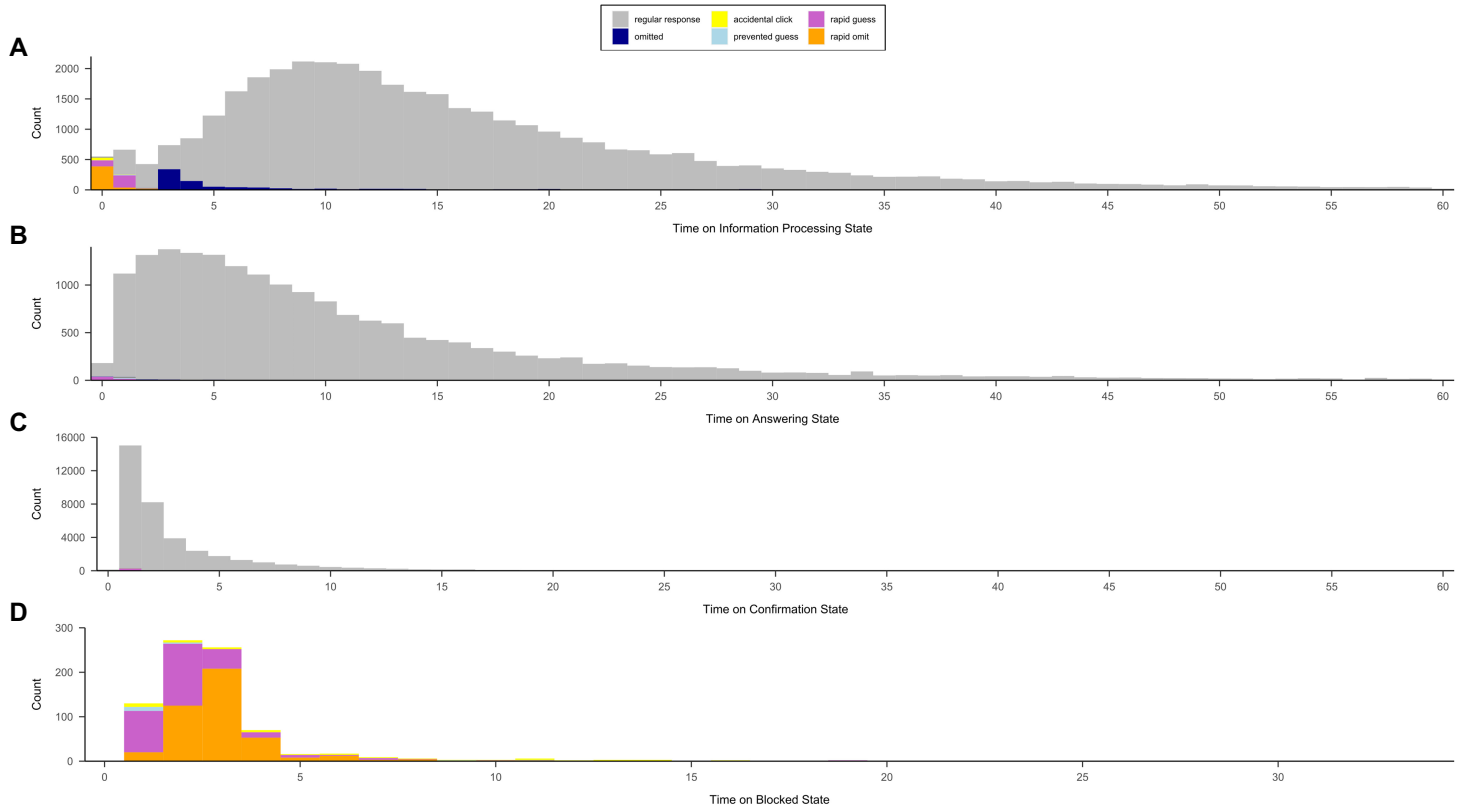
Response time in seconds per state of the response process and per response class (regular response, omitted, accidental click, prevented guess, rapid guess, rapid omit) for the low-stakes condition.

Figure 4 shows the times on state and the state-sequence class the response was assigned to for the low-stakes condition. A bimodal distribution is observable for the information processing state but not the other states. The first mode of the information processing distribution mainly constitutes of responses with ignored button clicks.

### Response time-based classification and intersection with state-sequence based classification

Two raters estimated the ChIA threshold values. There were no differences between raters for any of the 16 estimated thresholds. Table 3 shows the estimated response time threshold values per item type. The last column shows the number of responses classified as rapid responses through state-sequences, which have response times below the threshold value. Conversely, the proportion of responses below the ChIA threshold values also classified by their state-sequence class was 35.55% for the high-stakes data, and 41.97% for the low-stakes data.From the comparison of item-level versus item-type-level, using the NT10 method, there was a sensitivity of 0.91 for the item-type-level and a specificity of 0.99.

**Table 3:** Mean response times, threshold values per item type, percentage of overall classified responses, and percentage of responses from the non-returner group within classified responses.

Stakes	Test	Item type	Mean rt	Threshold	Percent classified	Percent classified no return
High	Vendor					
		Choice	43.73	2.25	0.02	18.42
		Sort	59.46	4.75	0.18	100.00
		Associate	61.85	5.75	0.19	-
		Imagemap	32.84	2.75	0.02	0.00
		Combined	45.08		0.04	19.51
	Mvmt					
		Choice	53.12	3.25	0.40	53.10
		Sort	74.28	5.75	2.21	100.00
		Associate	79.05	5.75	2.07	93.75
		Imagemap	37.50	2.75	0.00	0.00
		Combined	56.67		0.69	63.87
Low	Vendor					
		Choice	26.30	3.75	3.05	85.71
		Sort	34.81	4.25	1.41	87.50
		Associate	34.28	4.75	0.36	100.00
		Imagemap	17.41	2.25	0.47	27.27
		Combined	26.89		2.73	83.12
	Mvmt					
		Choice	24.53	4.25	5.16	91.58
		Sort	38.40	4.75	5.00	92.86
		Associate	40.02	4.75	5.44	83.87
		Imagemap	17.57	2.75	1.89	45.83
		Combined	26.87		5.05	89.13

Mvmt, motor vehicle mechatronics technician; rt, response time.

The “Combined” row shows mean response times, the percentage of overall classified responses, and percentage of responses from the non-returner group within classified responses across item types for each data set.

### Validation of response classification by state-sequences

#### Response times and accuracy

Mean scores and mean times of responses without ignored button click, of responses where participants returned to item interaction, and of responses where participants did not return are shown in Table 4. A manova with a tuckey *post hoc* analysis showed that all three groups differed significantly in response time and average scores for the low-stakes condition. For the high-stakes conditions, the response time of returners did not differ significantly for responses without ignored button clicks, but the score did. The number of observations was very small for the returner group. Because no significant difference between neither response time nor score of returners and regular responses was expected, it was checked whether the score would differ when controlled for response times. A generalized linear model was estimated to predict score by response time, and the groups returned, and regular response. The group remained a significant predictor under high-stakes conditions (z = 4.25, *p* < 0.001) as well as under low-stakes conditions (z = 8.37, p < 0.001).

**Table 4:** Time on task and mean score for responses without ignored button clicks, responses from the returner group, and responses from the non-returner group – *p*-values were estimated for a Tuckey *post hoc* test.

Stakes	Group	n	Response time (accepted)	Mean item-score	p time with previous	p score with previous
High						
	No ignored	164,309	47.73	0.57	0.000^***^	0.000^***^
	Returned	22	39.21	0.23	0.828	0.007^**^
	Not returned	196	4.69	0.01	0.000^***^	0.000^***^
Low						
	No ignored	38,463	27.42	0.64	0.000^***^	0.000^***^
	Returned	64	14.92	0.44	0.000^***^	0.001^**^
	Not returned	752	4.01	0.08	0.000^***^	0.000^***^

** *p* < 0.01;

*** *p* < 0.001.

#### Behavioral changes after blocked responses

In a first step, participants were ordered by the number of responses classified through the ChIA method. The biserial correlation between the number of classified responses with the ChIA method and having at least one response classified through state-sequences was r = 0.675. Hence, the more responses were classified through response time-based thresholds, the more likely a participant had responses classified through state-sequences. 30.36% of participants with more than one rapid response according to response time did not have a rapid response classified through state-sequences. One person from this group had 22, and one person had 15 classified responses. The remaining participants had 7 or fewer classified responses.

The next step was to check if participants with rapid guessing tendency stopped clicking while the forbidden sign was shown after experiencing the blocked response. Couplets of two rapid responses were formed within persons, flagged by response time or state-sequence class. The number of switches from rapid responses flagged by state-sequence and possibly response time to rapid responses flagged solely by response time was compared with switches oppositely. If participants learn to avoid clicking on the next button after a first encounter but continue giving rapid responses, more switches from responses flagged by state-sequence to responses solely flagged by response time were expected. Switches from responses flagged by state-sequences to responses flagged solely by response time (M = 2.11, SD = 2.58) were not more frequent than switches from responses flagged solely by response time to responses flagged by state-sequences (M = 2.04, SD = 2.55), t(224) = 0.21, *p* = 0.418.

**Figure 5:**
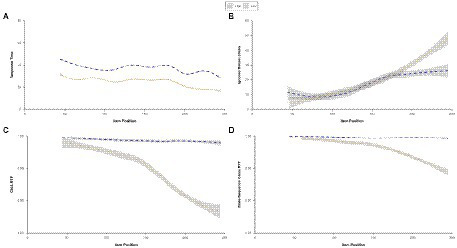
Mean response times, number of ignored button clicks, response time fidelity for ChIA, and state-sequence class by item position for the high- and low-stakes data.

The plot at the top left of Figure 5 shows response times in the course of the test. Response times are shorter for the low-stakes condition. For both conditions, response times decrease toward the end of the test. The plot at the top right shows the number of ignored button clicks increasing over time. The lower two plots from Figure 5 show RTF indicated by the ChIA method and state-sequence classes. Both RTF values decrease toward the end of the test in the low-stakes condition and show a minimal decrease after the start of the test in the high-stakes condition.

#### Covariates

##### Ability

The pearson correlation between ability estimates per area of expertise and RTE per area of expertise for the ChiA-method under high-stakes conditions was r (6190) = 0.23, *p* < 0.001 and r (1460) = 0.72, p < 0.001 under low-stakes conditions. The pearson correlation between ability and RTE for the state-sequence classes under high-stakes conditions was r (6190) = 0.07, *p* < 0.001 and r (1460) = 0.41, p < 0.001 under low-stakes conditions.

##### Gender

Regarding ChiA solution behavior, a chi-squared test turned out to be significant for high-stakes conditions, *χ*^2^ (1, *N* = 164,262) = 212.19, *p* < 0.001 as well as for low-stakes conditions, *χ*^2^ (1, *N* = 39,145) = 179.69, *p* < 0.001. Results indicate that female participants showed more often solution behavior than male participants. Regarding solution behavior according to the log data model, a chi-squared test turned out to be significant for high-stakes conditions, *χ*^2^ (1, *N* = 164,262) = 132.63, *p* < 0.001 as well as for low-stakes conditions, *χ*^2^ (1, *N* = 39,145) = 113.83, *p* < 0.001. Results indicate that female participants also showed more often solution behavior than male participants if state-sequences were used for classification.

##### Primary language

Regarding ChiA solution behavior, a chi-squared test turned out to be significant for high-stakes conditions, *χ*^2^ (1, *N* = 164,527) = 222.18, *p* < 0.001 as well as for low-stakes conditions, *χ*^2^ (1, *N* = 39,279) = 71.33, *p* < 0.001. Results indicate that German native speakers showed more often solution behavior than participants whose first language did not correspond to the test language. Regarding solution behavior according to the state-sequence classes, a chi-squared test turned out to be significant for high-stakes conditions, χ^2^ (1, N = 164,527) = 143.32, p < 0.001 as well as for low-stakes conditions, χ^2^ (1, N = 39,279) = 58.38, p < 0.001. Results indicate that German native speakers also showed more often solution behavior than participants whose first language did not correspond to the test language if state-sequences were used for classification.

##### Age

The mean age of participants in the high-stakes condition was *M* = 37.63 with *SD* = 11.32, while the mean age of participants under low-stakes conditions was *M* = 22.90 with *SD* = 6.78. The age in the low-stakes condition ranged from 18 to 58, but within the 90% decile, the maximum age was 25 years.

The pearson correlation between age and RTE for the ChiA-method under high-stakes conditions was *r* (1,244) = 0.01, *p* = 0.727, and *r* (301) = −0.23, *p* < 0.001 under low-stakes conditions. The pearson correlation between age and RTE for the state-sequence classes under high-stakes conditions was *r* (1,244) = 0.16, *p* < 0.001, and *r* (301) = −0.21, *p* < 0.001 under low-stakes conditions.

## Discussion

Taken together, the finite-state machine approach helped derive interpretable response classes from the accumulated log data. Evidence for the interpretation could be obtained based on falsifiable assumptions and allowed for a better understanding of the effect of a blocked response on the response process. All six predefined classes could be found empirically. The prevented guess class was the less frequent class, with only a few cases in both data sets. Accidental clicks were also scarce. Most of the responses with ignored button clicks were from the rapid guess and rapid omission class, concluding that the blocked item response seems to be related chiefly to rapid responding. Responses with ignored button clicks could be found for almost all items. All response time-based thresholds were above 2 s. Consequently, it can be assumed that the 2 s blocking period is too short to expect responses within that time frame to represent solution behavior. It is possible that the coarsening on item type level has masked smaller thresholds. The sensitivity of the NT10 on item-type level indicates that the coarsening is acceptable and the items are sufficiently homogenous on the item-type level. The specificity was unsurprisingly high regarding the low amount of rapid responses. Nonetheless, with a prolonged blocking time (e.g., 3 s and more), fast responses with ignored button clicks could also represent fast engaged responses. Minimal responding times should be determined for high-performers in test construction. A recommendation for implementing a blocked item response would be that it should not exceed the minimal time needed for information processing. Conversely, the time span does not need to capture all quick responses since the preventive function is not dominant. For the *post hoc* detection, 2 s did capture a sufficient number of rapid responses. Another point is that the timer should be implemented in a way that takes into account possible technical problems such as slow loading of content. Some participants may wait until the loading process is complete before guessing or skipping.

An ancillary finding of this study is that the bimodal response time distribution seems to stem from the information processing state. No bimodal time on state distribution was found for the answering state or the response confirmation state. Solution behavior did start immediately. This could indicate that little time between selecting multiple answers is not a sign of disengagement, and neither would be quick response confirmation. It would be interesting if this finding could be replicated and found in more complex item formats.

Regarding the validity of the response time classes, rapid responses from the rapid guess and rapid omission class had significantly shorter response times and lower scores than responses without ignored button clicks and responses from the accidental click and the prevented guess class. This is consistent with the expectation that rapid guessing and rapid omission correspond to disengagement, whereas accidental clicking and prevented guessing correspond to solution behavior. Responses from the accidental click and prevented guesses class did differ in score and for the low-stakes conditions also in mean times from responses without ignored button clicks. However, mean times and mean scores were in the range of solution behavior. An explanation could be that accidental clicks are more likely if participants are inexperienced with computers and that the computer-based test format is also more difficult for those participants. Inexperience with computers is rather unlikely within the younger sample of vocational students. An alternative explanation could be that participants who prefer speed over accuracy are more prone to experience accidental clicks. Nevertheless, due to the small number of responses, the results should be interpreted cautiously, and further investigations are needed.

Another aspect for the validity of the state-sequence classes was that some participants avoided clicking on the next button, while the mouse-courser showed a forbidden sign. Only two cases with a higher number of rapid responses classified through response time thresholds could be found that did not have ignored button clicks in the log data. It is unclear if they reacted to the forbidden sign from the start or had longer reaction times when answering quickly. Mouse coordinates were not included in the data set but could provide information in future studies. Besides those cases where participants consequently did not produce ignored button clicks while giving rapid responses, no decrease in ignored button clicks in relation to rapid responses could be found in the course of the test. Participants did not seem to adapt their behavior as a consequence of the encounter with the 2-s-blocking.

Finally, the results showed a relation of the covariates ability, gender, and primary language to rapid responding as expected from former studies (DeMars et al., 2013; Setzer et al., 2013; Goldhammer et al., 2016, 2017). This can be seen as evidence that the state-sequence classes and the ChIA method proved valid in identifying disengagement. The findings were consistent across high-and low-stakes conditions, with the expected difference that rapid responding was less frequent under high-stakes conditions. The correlation between ability and rapid responses was larger for the state-sequence based classification compared to the ChIA method. This could be interpreted as either that the ChIA method is less valid or that ignored button clicks are more common among individuals with low ability and higher-skilled individuals avoid the blocked response. Investigating differential functioning of the blocked response would be of interest to further research. Age did show a small negative relation for the low-stakes conditions, which was not expected, but is difficult to interpret due to the low variance within the sample. However, the negative relation under low-stakes conditions was found for the state-sequence classes and the ChIA, which indicates no age effects for the probability of ignored button clicks. The small positive relation between RTE and age for the state-sequences that could not be found for the ChiA method could indicate some age effects like prolonged reaction times that reduced the probability for ignored button clicks. But the explained variance is marginal. The results also indicate that response behavior was affected by item features. Besides the score, which relates to item difficulty and thus ability and item length, item format could be more or less promoting for certain behavior. Imagemaps showed relatively more rapid omissions than rapid guesses compared to choice items, although they followed in effect a multiple choice principle. It also seems reasonable that item design can be more or less appealing and thus capture attention and encourage engagement.

### Limitations and future research

A question regarding the blocked state in the response process is what cognitive processes it could be associated with. Is the blocked state the time it takes to make the decision to continue the rapid response or to return to item interaction? This could as well be a multi-stage process where the participant takes a look at the item but then again decides against solution behavior. In an experimental setting, future research could use interviews to get more insights or use more subtle techniques like eye-tracking to see if participants look again at the question.

A benefit of the 2-s-blocking is that it offers the potential to classify rapid responses at the intersection of the response time distribution of rapid responses and solution behavior. Proportions correct, and the change in information used in the ChIA method try to capture the onset of the solution behavior distribution. Regarding the empirical findings, response times and proportions correct do indicate that rapid responses with no interactions after the ignored button click do not represent solution behavior but disengaged rapid responding instead. Therefore, the response times of accepted responses from the non-returner group could be used for a heuristic approach to determine the response time distribution of rapid responses in general. This distribution could be included in visual inspection methods, as shown in Figure 2.

### Conclusion

In summary, the 2-s-blocking intended effect of preventing accidental clicks could only be found for a small proportion of the responses. Prevented guessing plays a negligible role. In most cases, ignored button clicks could be associated with rapid responding. However, rapid responding rates were relatively low in the data. Nevertheless, even though accidental clicks were less frequent than rapid responses, they did occur, and blocking the response button for a short amount of time did work as a countermeasure. Especially in high-stakes assessments, this could be reason enough for test designers to include response blocking. If response times are used to detect disengagement in an assessment with such a feature, log events from item interactions and button clicks are necessary for proper classification. Not only the count but also the sequence of log events is relevant. The finite-state approach proved helpful in distinguishing different behavioral patterns by analyzing state-sequences. The findings were consistent across conditions and test progress. Furthermore, log data of rapid responses with blocked responses can be used as a heuristic for the upper limit of response times from rapid responses. This heuristic can help to identify rapid responses without blocked item responses. Overall blocking the item response did not show an effect in preventing disengaged response behavior. But in combination with log data, it did prove helpful in detecting it.

## Data availability statement

The data analyzed in this study is subject to the following licenses/restrictions: scientific use. Requests to access these datasets should be directed to https://fdz.iab.de/.

## Ethics statement

Ethical review and approval was not required for the study on human participants in accordance with the local legislation and institutional requirements. Written informed consent from the patients/ participants or patients/participants legal guardian/next of kin was not required to participate in this study in accordance with the national legislation and the institutional requirements.

## Author contributions

All authors listed have made a substantial, direct, and intellectual contribution to the work and approved it for publication.

## Conflict of interest

The authors declare that the research was conducted in the absence of any commercial or financial relationships that could be construed as a potential conflict of interest.

## Publisher’s note

All claims expressed in this article are solely those of the authors and do not necessarily represent those of their affiliated organizations, or those of the publisher, the editors and the reviewers. Any product that may be evaluated in this article, or claim that may be made by its manufacturer, is not guaranteed or endorsed by the publisher.

## Footnotes

^1^Due to the circumstance that participants in the regular operations may not have experience in all areas of expertise, they are advised to skip the area they were not familiar with by omitting all items and proceed with the next area of expertise. In the present data, results of two participants from the regular operations had ignored button clicks in more than 90% of the items and were excluded from the analysis. This advice was not given to the standardization sample.
